# Optimizing Urological Concurrent Robotic Multisite Surgery: Juxtaposing a Single-Center Experience and a Literature Review

**DOI:** 10.3390/jpm14101053

**Published:** 2024-10-11

**Authors:** Rafał B. Drobot, Marcin Lipa, Weronika A. Zahorska, Daniel Ludwiczak, Artur A. Antoniewicz

**Affiliations:** 1 Urology Department, Institute of Medical Sciences, Faculty of Medicine, Collegium Medicum, Cardinal Stefan Wyszyński University in Warsaw, Bursztynowa St. 2, 04-479 Warsaw, Poland; m.lipa@uksw.edu.pl (M.L.); w.zahorska@uksw.edu.pl (W.A.Z.); a.antoniewicz@uksw.edu.pl (A.A.A.); 2 Department of Urology and Urological Oncology, Multidisciplinary Hospital in Warsaw-Miedzylesie, Bursztynowa St. 2, 04-479 Warsaw, Poland

**Keywords:** robotic surgery, robot-assisted partial nephrectomy, robot-assisted radical prostatectomy, concurrent multisite surgery, synchronous prostate and kidney cancers

## Abstract

**Introduction**: This article juxtaposes case series with a systematic review to evaluate the feasibility, safety, and clinical outcomes of concurrent robotic multisite urological surgeries, specifically robot-assisted radical prostatectomy (RARP) and robot-assisted partial nephrectomy (RAPN), for synchronous prostate and kidney cancers. **Aim**: The aims of this study were to evaluate the feasibility, safety, and clinical outcomes of urological concurrent robotic multisite surgeries through a comparison of institutional findings with the existing literature. **Materials and Methods**: A retrospective analysis was conducted on eight institutional cases of concurrent robotic multisite surgeries performed between 2021 and 2024. The primary outcomes measured were operative time, blood loss, and postoperative complications. A systematic review of the literature was performed, searching PubMed, Embase, and Cochrane Library databases, with the last search conducted on 1 July 2024. Studies were included if they reported on concurrent robotic surgeries corresponding to the procedures performed at the institution, including RARP with RAPN, RARP with robotic transabdominal preperitoneal inguinal hernia repair (RTAPPIHR), and other multisite robotic surgeries. Risk of bias was assessed using the modified Newcastle–Ottawa Scale. Descriptive statistics were used to analyze operative time and blood loss, with confidence intervals (CIs) calculated to assess precision. Categorical variables, including postoperative complications, were summarized using frequencies and percentages. Heterogeneity was assessed using the I^2^ statistic, with values above 50% indicating substantial heterogeneity. A random effects model was applied when necessary, and sensitivity analyses excluded studies with high risk of bias. **Results**: We describe a unique docking technique employed in our procedures, which allows for atraumatic transitions between surgeries using the same port sites. Our institutional cases demonstrated the feasibility and safety of concurrent robotic multisite surgery, with a mean operative time of 315 min (95% CI: 290–340) and mean blood loss of 300 mL (95% CI: 250–350). There were no significant intraoperative complications reported. These findings are consistent with the literature, where mean operative times range from 390 to 430 min and blood loss ranges from 200 to 330 mL. Notably, no positive surgical margins or declines in postoperative renal function were observed in our cases. The systematic review included nine retrospective studies involving 40 cases of concurrent RARP and RAPN, as well as eleven studies including 392 cases of RARP combined with RTAPPIHR. The findings from these studies support the feasibility and safety of concurrent surgeries, showing similar rates of operative time, blood loss, and postoperative complications. **Conclusions:** Concurrent robotic multisite surgeries, such as RARP combined with RAPN or RTAPPIHR, appear to be safe and feasible. Our data suggest these procedures are non-inferior to separate surgeries in terms of safety and complication rates. Potential benefits, including reduced operative times, shorter hospital stays, and more efficient resource use, may translate into cost savings, although no formal cost-effectiveness analysis was conducted. Limitations include the small sample size, retrospective design, and lack of long-term follow-up. Prospective trials are needed to validate these findings and further refine the techniques. **Funding:** this review did not receive any external funding. **Registration:** this review was not registered in any public protocol registry due to its comparative retrospective nature.

## 1. Introduction

Robotic surgery revolutionized the field of urology, offering enhanced precision and minimally invasive options for complex surgical procedures. The concurrent execution of robot-assisted radical prostatectomy (RARP) and robot-assisted partial nephrectomy (RAPN) is an innovative approach to managing synchronous primary cancers of the prostate and kidney. This combined procedure leverages the advantages of robotic systems to perform two major surgeries in a single setting, reducing patient morbidity and healthcare costs associated with separate operations.

Reports in the literature indicate that single-setting robotic surgeries for both conditions are feasible and safe, providing benefits such as reduced abdominal trauma, shorter recovery times, and lower overall hospitalization costs [1,2,3,4]. This is significant as the increasing use of prostate cancer screening and cross-sectional imaging led to increases in the incidental detection of synchronous renal tumors in patients with diagnosed and staged prostate cancer [1,2]. However, combining these surgical procedures requires meticulous surgical planning and advanced expertise in robotic surgery.

Inguinal hernias are common in patients undergoing RARP. Traditionally, hernias are treated separately from prostate cancer surgeries. However, advancements in robotic surgery made it possible to combine RARP with robotic transabdominal preperitoneal inguinal hernia repair (RTAPPIHR), offering a comprehensive, single-session approach to managing both conditions.

This article presents an institutional case series of urological concurrent robotic multisite surgery from the Urology Department, Institute of Medical Sciences, Faculty of Medicine, Collegium Medicum, Cardinal Stefan Wyszyński University in Warsaw alongside a comprehensive literature review. It focuses specifically on concurrent RARP and RAPN surgeries performed using the da Vinci X robotic system with dual-console capabilities.

## 2. Aims

This study aims to evaluate the feasibility, safety, and clinical outcomes of concurrent robotic multisite surgery in urological patients. By presenting an institutional case series and reviewing the corresponding literature, this study seeks to demonstrate the efficacy of simultaneous robotic procedures, specifically focusing on unique docking techniques, surgical approaches, and short- and long-term outcomes. Additionally, by comparing the institutional findings with the existing literature, it highlights the advantages, challenges, and innovations of these combined surgical procedures. Through a detailed analysis of operative times, blood loss, complications, and postoperative outcomes, this study aspires to provide valuable insights that could enhance surgical practices and patient care in urological oncology.

In this context, the primary endpoints are centered on assessing the feasibility and safety of the procedures, including key metrics such as operative time, estimated blood loss, and the incidence of perioperative complications. The secondary endpoints involve evaluating long-term clinical outcomes, with a particular emphasis on the preservation of renal function (as measured by changes in estimated glomerular filtration rate), the presence of positive surgical margins, and the duration of hospitalization. The outcomes of interest encompass both intraoperative and postoperative variables, allowing for a detailed understanding of the surgical approach’s effectiveness in managing complex urological conditions. The overarching research question sought to determine the clinical efficacy and potential benefits of concurrent robotic procedures in urological patients, particularly in optimizing surgical workflow, patient recovery, and minimizing complications.

## 3. Material and Methods

### 3.1. Patient Selection for the Institutional Case Series

**Figure 1:**
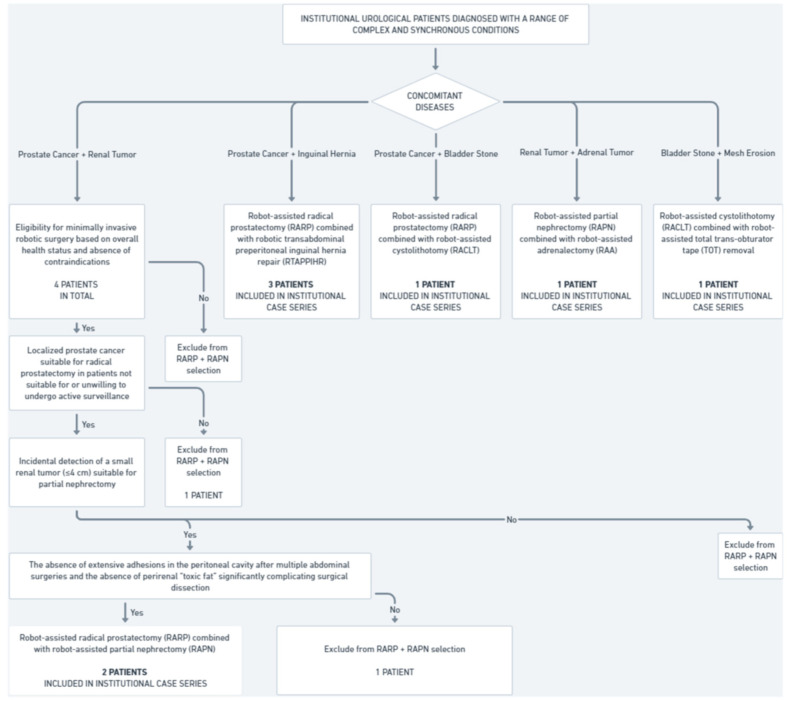
Institutional cohort selection for concurrent robotic multisite surgeries (2021–2024).

This study includes a retrospective analysis of eight patients treated at the Urology Department, Institute of Medical Sciences, Faculty of Medicine, Collegium Medicum, Cardinal Stefan Wyszyński University in Warsaw who were subjected to concurrent robotic multisite surgery (Figure 1).

Between 2021 and 2024, four patients were diagnosed with synchronous prostate cancer and small renal tumors; two of them underwent concurrent robot-assisted radical prostatectomy (RARP) and robot-assisted partial nephrectomy (RAPN). The inclusion criteria were as follows:

1. Confirmed diagnosis of localized prostate cancer suitable for radical prostatectomy in patients not suitable for or unwilling to undergo active surveillance.
2. Incidental detection of a small renal tumor (≤4 cm) suitable for partial nephrectomy.
3. Eligibility for minimally invasive robotic surgery based on overall health status and absence of contraindications.
4. The absence of extensive adhesions in the peritoneal cavity after multiple abdominal surgeries and the absence of perirenal “toxic fat” significantly complicating surgical dissection.

The two other patients did not meet these criteria. As a result, they were subjected to non-simultaneous robot-assisted surgical treatment and were, therefore, not part of our eight-patient case series.

During the same time frame, three patients were diagnosed with synchronous prostate cancer and inguinal hernia, and all of them underwent concurrent robot-assisted radical prostatectomy and robotic transabdominal preperitoneal inguinal hernia repair (RTAPPIHR).

Additionally, during the same period, three other patients underwent various combinations of robot-assisted surgeries, including partial nephrectomy, adrenalectomy, total trans-obturator tape (TOT) removal, cystolithotomy, and radical prostatectomy, demonstrating the capability and versatility of robotic systems in managing complex multiorgan surgical interventions within a single operative session. In total, eight institutional patients were included in the analysis of concurrent robotic multisite surgeries, comprising the entire population that underwent simultaneous robot-assisted procedures in our center over the past four years.

### 3.2. Data Collection for the Institutional Case Series and the Literature Review

Data, if available, were collected retrospectively from patient medical records pertaining to cases treated by concurrent robotic multisite surgery at our institution, as well as from relevant studies published in English and retrieved in full-text versions from the PubMed, Embase, and Cochrane Library databases. The present review was conducted in accordance with the Preferred Reporting Items for Systematic Reviews and Meta-Analyses (PRISMA) 2020 statement. However, the study protocol was not registered with PROSPERO or any other protocol registry before the review was initiated, due to its retrospective comparative design, which does not align with PROSPERO’s criteria for registration.

### 3.3. Search Strategy

To ensure a comprehensive review of the literature, a structured search strategy was employed using specific keywords and Medical Subject Headings (MeSH) terms. The last search was performed on 1 July 2024. No limitations were imposed on time. Boolean operators were utilized to combine these keywords effectively. No automation tools were used in the screening or selection process. Two review authors (RBD and ML) independently conducted a comprehensive literature search across three electronic databases using the following search string:

○ For concurrent RARP + RAPN: (“concurrent” OR “simultaneous” OR “combined” OR “multivisceral”) AND (“robot-assisted” OR “robotic”) AND (“prostatectomy” AND “partial nephrectomy”) OR (“resection”).
○ For concurrent RARP + RTAPPIHR: (“concurrent” OR “concomitant”) AND (“robot-assisted” OR “robotic”) AND (“prostatectomy” OR “radical prostatectomy” OR “RALP” OR “RARP”) AND (“hernia” OR “inguinal hernia repair” OR “IHR”).
○ For other combinations of robot-assisted concurrent multisite surgeries: (“robot-assisted” OR “robotic”) AND (“stone” OR “cystolithotomy” OR “partial nephrectomy”) AND (“radical prostatectomy” OR “mesh removal” OR “adrenalectomy”).

### 3.4. Inclusion and Exclusion Criteria

The eligibility of retrieved studies was evaluated using the population, intervention, comparison, outcome, and study design (PICOS) approach. The criteria for inclusion in the present comprehensive review were as follows:

○ (P)opulation: Institutional urological patients diagnosed with a range of complex and synchronous conditions, including:
  ▪ Prostate cancer with concurrent small renal tumors.
  ▪ Prostate cancer with concurrent inguinal hernia.
  ▪ Prostate cancer with concurrent bladder stones.
  ▪ Bladder stones with concurrent mesh perforation (after trans-obturator tape procedures).
  ▪ Kidney tumors with concurrent adrenal gland involvement.
○ (I)ntervention:
  ▪ RARP combined with RAPN.
  ▪ RARP combined with RTAPPIHR.
  ▪ RARP combined with robot-assisted cystolithotomy (RACLT).
  ▪ RACLT combined with robot-assisted total trans-obturator tape removal.
  ▪ RAPN combined with robot-assisted adrenalectomy (RAA).
○ (C)omparison: data from the comprehensive literature review of studies involving procedures analogous to the concurrent robotic multisite surgeries performed at our institution.
○ (O)utcome: The collected data include the following:
  ▪ Operative time (total surgery time and console time for each separate procedure, if available).
  ▪ Estimated blood loss.
  ▪ Perioperative complications.
  ▪ Pathological outcomes, including biopsy results and post-prostatectomy histopathology in Gleason scores, as well as post-partial nephrectomy histopathology.
  ▪ Hemoglobin levels pre- and post-operation.
  ▪ Estimated glomerular filtration rate (eGFR) levels pre- and post-operation (24 h after surgery if available).
  ▪ Hospitalization period.
  ▪ Indications for surgery (if needed to be explained).
○ (S)tudy design: retrospective cohort studies, case series, and single-center experiences.

Reference lists of identified articles were also manually searched to ensure that no relevant studies were missed. This method allowed for a systematic and reproducible approach to the literature search.

All identified articles were thoroughly reviewed, and only studies that provided detailed and relevant clinical or perioperative data pertinent to the outcomes of interest were included in the analysis.

### 3.5. Quality Assessment and Risk of Bias for the Review of the Literature

Each study included in the review was subjected to a rigorous quality assessment and risk of bias (RoB) evaluation using a modified version of the Newcastle–Ottawa Scale (NOS). This scale evaluates the quality of non-randomized studies based on selection, comparability, and outcome assessment. Studies scoring below a predetermined threshold of 8 points were excluded to ensure that only high-quality evidence was included in the final analysis. The RoB of each study was assessed independently by two authors (RBD and ML). Disagreements were resolved through consultation with the senior author (AAA). This quality assessment framework ensures that the review’s conclusions are based on robust and reliable data, enhancing the credibility and applicability of the findings to clinical practice. The detailed data on the quality assessment of individual studies using the modified Newcastle–Ottawa Scale are presented in Table 1 for studies involving robot-assisted radical prostatectomy (RARP) and partial nephrectomy (RAPN) and in Table 2 for studies involving robot-assisted radical prostatectomy (RARP) and transabdominal preperitoneal inguinal hernia repair (RTAPPIHR). Table 3 presents the NOS for other corresponding concurrent urological robotic multisite surgery procedures.

### 3.6. Data Extraction

Two review authors (RBD and ML) separately extracted a predefined set of data from the included studies using standard data extraction templates. Throughout the data extraction process, any inconsistencies were resolved through consultation with a senior co-investigator (AAA). The certainty of the evidence for each outcome was assessed using the GRADE approach. Factors such as risk of bias, inconsistency, indirectness, imprecision, and publication bias were considered when evaluating the strength of the evidence. To assess the risk of reporting bias, we evaluated whether the included studies reported all pre-specified outcomes. We did not detect substantial evidence of selective reporting, although the absence of full-text access to several studies could have introduced a potential reporting bias.

### 3.7. Statistical Analysis

All statistical analyses were performed using Statistica 13.3 (StatSoft Inc., Tulsa, OK, USA). Descriptive statistics were used to summarize the data. For continuous quantitative variables, such as operative time, estimated blood loss, hemoglobin levels, and eGFR levels, arithmetic means were calculated to provide the central tendency of the data. For categorical qualitative variables, such as perioperative complications and pathological outcomes, frequencies and percentages were used to describe the distribution of these events. We synthesized the results by comparing the key metrics (operative time, blood loss, and complications) across the studies. Heterogeneity was assessed using the I^2^ statistic, and an I^2^ value above 50% was considered indicative of substantial heterogeneity. A random-effects model was applied where significant heterogeneity was present. Sensitivity analyses were conducted by excluding studies with high risk of bias, ensuring robustness of the synthesized results.

In this study, confidence intervals (CIs) were calculated to evaluate the precision of key outcomes, such as operative time, blood loss, and changes in the glomerular filtration rate. These intervals are visually represented as error bars in the figures, where the mean values for each variable are shown, making it easy to assess the precision and variability of the results across different studies and institutional data.

This approach provided a comprehensive overview of the data, which was essential for comparing institutional outcomes with the existing literature and enabling a robust assessment of the feasibility, effectiveness, and safety of concurrent procedures.

### 3.8. Ethical Considerations

This study was conducted in accordance with the ethical standards of the institutional and national research committees and with the 1964 Declaration of Helsinki and its later amendments. Informed consent was obtained from all individual participants included in the study.

## 4. Results

### 4.1. Unique Docking Technique in Our Concurrent RARP + RAPN Procedures Using the Same Port Sites

**Figure 2:**
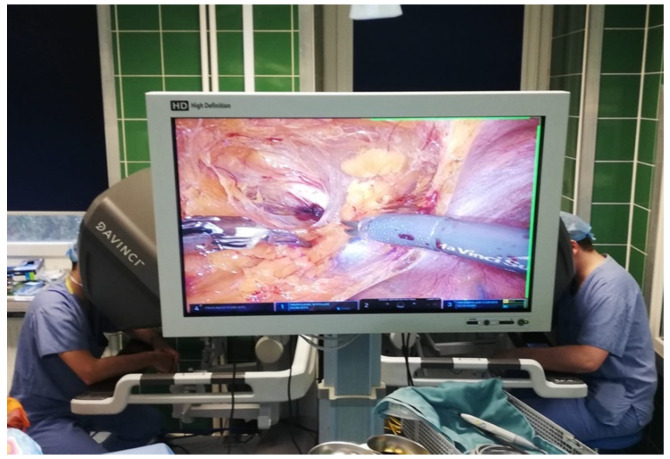
Dual-console robotic surgery.

All surgeries were performed using the da Vinci X robotic system with dual-console capabilities (Figure 2). The surgical team consisted of two experienced robotic surgeons. The procedures were carried out under general anesthesia, with the patient in different positions for each procedure.

**Figure 3:**
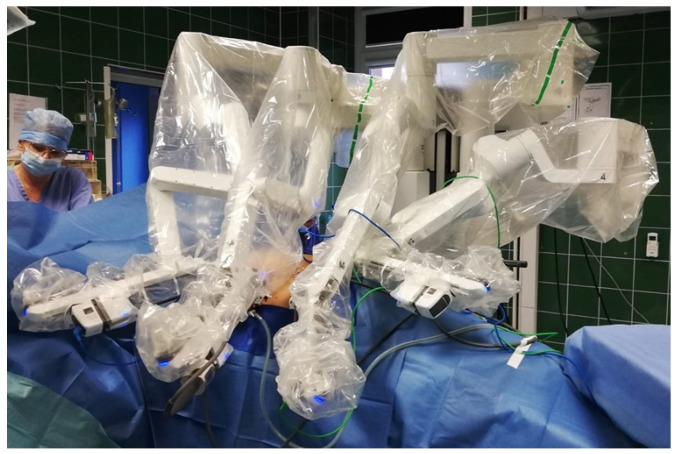
Patient in the lateral decubitus position during robot-assisted partial nephrectomy (RAPN).

**Figure 4:**
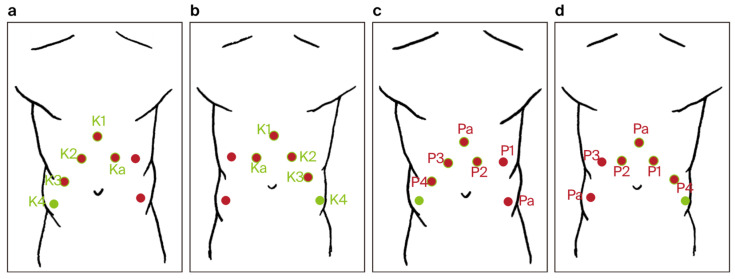
Trocar port placement for the combined robot-assisted radical prostatectomy (RARP) + robot-assisted partial nephrectomy (RAPN) procedure (**a**–**d**): red dots—trocar sites used only during RARP, green dots—trocar sites used only during RAPN, red dots with green rim—trocar sites common to both RARP and RAPN, P1–P4—trocar sites for robotic arms during RARP, K1–K4—trocar sites for robotic arms during RAPN, Pa—trocar sites for the assistant during RARP, and Ka—trocar sites for the assistant during RAPN.

1. Robot-Assisted Partial Nephrectomy (RAPN):
  ○ The patient was initially placed in the lateral decubitus position (Figure 3).
  ○ One 12 mm laparoscopic trocar (assistant trocar) and four 8 mm robotic trocars were used (a total of five).
  ○ The renal artery was isolated, clamped during the tumor resection, and unclamped after renorrhaphy.
  ○ The renal tumor was excised with a margin of healthy tissue.
  ○ Renal reconstruction (renorrhaphy) was performed using a two-layer closure technique:
    ▪ The inner layer was closed using a 3–0 monofilament suture on a 26 mm needle.
    ▪ The outer layer (fibrous capsule and tumor bed) was closed using a barbed 3–0 V-lock™ suture on a 26 mm needle, with Hem-o-lock™ clips and TachoSil™ hemostatic material placed under the outer sutures.
  ○ After decompression of the renal artery, hemostasis was verified at the pressure of a 6 mm column of mercury inside the peritoneal cavity.
  ○ The kidney tumor was pulled out in an Endo Bag™ with the assistant’s trocar.
2. Repositioning:
  ○ After the completion of RAPN, the robotic system was undocked.
  ○ Arm 4 switched position to the opposite side in the da Vinci X robotic system.
  ○ The patient was repositioned to the supine Trendelenburg position at a 30-degree angle, and the robotic system was re-docked for RARP.
3. Robot-Assisted Radical Prostatectomy (RARP):
  ○ Trocar placement for RARP was modified by using the previous incisions from the RAPN procedure. Four 8 mm robotic trocars and two 11 mm laparoscopic trocars were used (a total number of six) (Figure 4).
  ○ The fourth robotic arm port was closed, and two new incisions were made: one for a laparoscopic trocar and one for a robotic trocar.
  ○ The key steps included the dissection of the prostate with bladder neck sparing when possible, control of the dorsal venous complex, nerve-sparing techniques when applicable, and vesicourethral anastomosis with a continuous double-needle suture.
  ○ A bladder–urethral anastomosis leak test of 300 mL in the bladder was performed.
  ○ The prostatectomy specimen was pulled out with the assistant’s trocar in the Endo Bag™.
  ○ Only one 18 Ch Redon drain was inserted into the peritoneal cavity after the combined procedure.
4. Robotic Instruments
  ○ The same robotic instruments were used for both procedures:
    ▪ Large needle driver.
    ▪ ProGrasp forceps.
    ▪ Monopolar curved scissors.
    ▪ Fenestrated bipolar forceps.

### 4.2. Search Results

**Figure 5:**
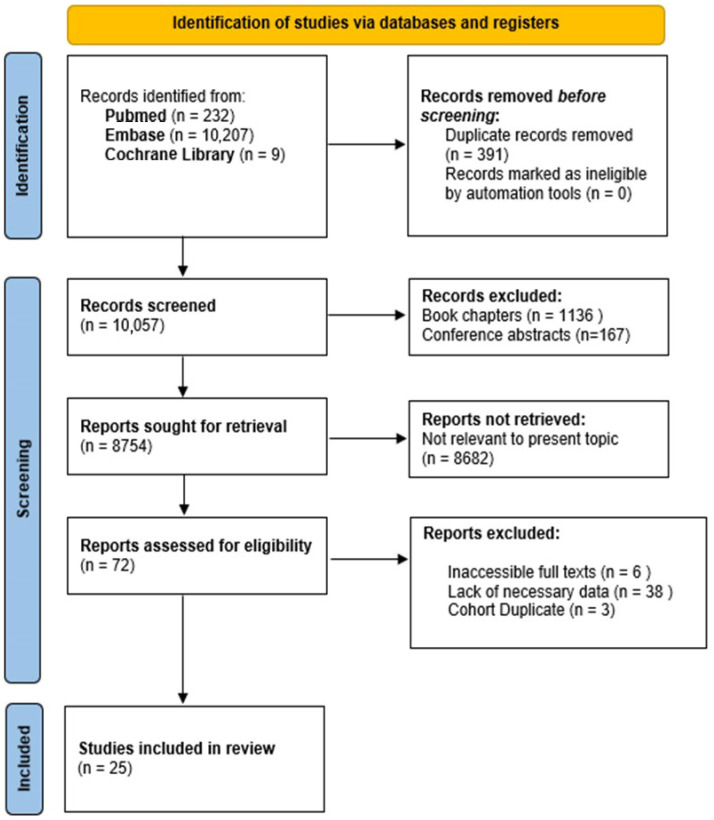
Diagram illustrating the study selection process following PRISMA guidelines.

Figure 5 provides a comprehensive overview of the study selection process. The initial systematic literature search yielded 10,448 publications from PubMed, Embase, and the Cochrane Library. After the removal of 391 duplicate records, 10,057 articles were screened. During the title and abstract screening, 1136 book chapters and 167 conference abstracts were excluded. Of the 8754 reports considered for full-text review, 8682 were excluded as irrelevant to the topic. Additionally, 47 full-text articles were removed due to inaccessibility (6), lack of necessary data (38), or duplicate cohorts (3). Ultimately, 25 studies were included in the review.

### 4.3. Overview

The outcomes documented in this study offer a thorough analysis of robotic multisite surgical procedures conducted at our institution, and detailed data are provided in Table 4, Table 5, Table 6 and Table 7.

Table 4 focuses on simultaneous procedures involving robot-assisted radical prostatectomy (RARP) and robot-assisted partial nephrectomy (RAPN), highlighting key clinical outcomes, including operative time, estimated blood loss, and postoperative complications. Table 5 examines cases where RARP and RAPN were performed at different intervals, offering insights into the surgical approach and patient recovery associated with staged operations. Table 6 delves into the integration of RARP with robotic transabdominal preperitoneal inguinal hernia repair (RTAPPIHR), focusing on perioperative outcomes and complication rates, while Table 7 encapsulates the results from other complex, concurrent robotic interventions carried out at our facility.

To contextualize our institutional findings, we compared them with data from the existing literature on robotic multisite surgery, as synthesized in Table 8, Table 9 and Table 10.

Table 8 contrasts our concurrent RARP and RAPN outcomes with those reported globally, evaluating parameters such as operative time, blood loss, and renal function preservation. Table 9 aggregates results from studies on the combination of RARP with robot-assisted inguinal hernia repair, juxtaposing them against our institutional experience. Finally, Table 10 reviews additional concurrent robotic procedures analogous to those performed in our institution, offering a comparative perspective on the safety and feasibility of these complex surgeries.

### 4.4. Cumulative Analysis

**Figure 6:**
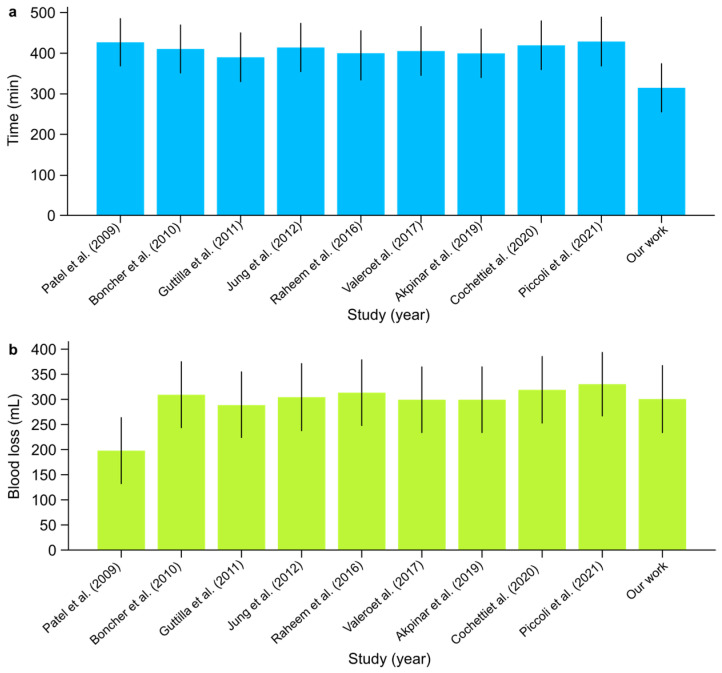
(**a**) Mean operative time (min) and (**b**) mean estimated blood loss (mL) with confidence intervals among scientific reports [1,2,3,4,5,6,7,8,9] on concurrent RARP + RAPN.

I. Concurrent RARP + RAPN
  1. Operative Time:
    ○ The mean operative time across studies ranged from 390 to 430 min. In comparison, our institutional study reported the shortest mean operative time of 315 min, demonstrating non-inferiority in time efficiency and highlighting a potentially improved surgical workflow (Figure 6). The overall certainty of the evidence for operative time was rated as low due to the very high heterogeneity observed across studies (I^2^: 99.91%). Despite this, the mean value of 400.7 min (95% CI: 377.19–424.21) was consistent with prior findings, though the extreme heterogeneity limits the precision of this estimate.
  2. Console Time:
    ○ Console times varied between 250 and 335 min across the studies. Our institutional data show a console time of 270 min, which fell within this range and indicated consistent performance. The certainty of the evidence for console time was rated as moderate, despite the high heterogeneity (I^2^: 90%), indicating substantial variability between studies. The mean console time of 275 min (95% CI: 257.72–292.28) aligns with the reported range, but variability across centers affects the robustness of this estimate.
  3. Estimated Blood Loss:
    ○ Blood loss was generally low across studies, averaging between 200 and 330 mL. Our study reported an estimated blood loss of 300 mL, which was consistent with the range observed in other studies (Figure 6). The certainty of the evidence for estimated blood loss was rated as low, given the very high heterogeneity (I^2^: 99.92%) observed across studies. The mean blood loss of 297 mL (95% CI: 271.25–322.75) was within the expected range, but the extreme variability between institutions reduces the confidence in this estimate.
  4. Complications:
    ○ None of the reviewed studies, including ours, reported significant perioperative complications (Clavien–Dindo), confirming the overall safety of the procedure.
  5. Positive Surgical Margins:
    ○ While positive surgical margins were observed in a small percentage of cases across various studies, none were reported in our study, highlighting the precision of our surgical technique.
  6. Renal Function:
    ○ The postoperative estimated glomerular filtration rate (eGFR) generally showed a slight decline immediately but had stabilized by the one-month follow-up in most studies, with values ranging between −4 and −5 mL/min/1.73 m^2^. Notably, our study showed an increase in eGFR of +24.85 mL/min/1.73 m^2^, indicating an exceptional renal function outcome compared with the other studies. The certainty of the evidence for the difference in eGFR before and after surgery was rated as low due to the significant heterogeneity (I^2^: 85%) and limited sample size. The mean difference of −1.46 mL/min/1.73 m^2^ (95% CI: −8.53–5.60) reflects substantial variation, limiting the generalizability of the findings. Comprehensive postoperative care, including meticulous management of hydration and renal perfusion, can aid in the recovery of renal function.
  7. Hospitalization:
    ○ The length of hospital stay varied between 2 and 8 days in the reviewed studies. Our institutional study reported a hospitalization time of 5.5 days, which was within this range and suggested comparable postoperative recovery times. The certainty of the evidence for hospitalization time was rated as moderate, with an I^2^ value of 70%, indicating substantial variability between institutions. The mean hospitalization time of 6.05 days (95% CI: 4.85–7.25) was consistent with prior reports, though further standardization of care could help reduce this variability.

Summary

Our study showed non-inferiority in terms of operative time and a notable increase in postoperative eGFR compared with other studies, while maintaining consistent console times, blood loss, complication rates, and hospitalization durations. Despite the observed substantial heterogeneity across studies, the overall certainty of the evidence for most metrics, such as operative time and blood loss, was rated as low, reflecting the high variability between studies. These results highlight the safety, efficiency, and effectiveness of our surgical approach, particularly in renal function outcomes, which are consistent with the findings reported in the current literature. Further standardization of care may help reduce variability and improve the robustness of the outcomes.

**Figure 7:**
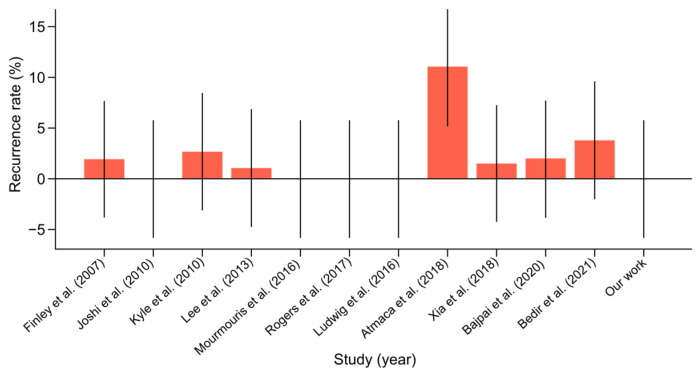
Hernia recurrence rate with confidence intervals among scientific reports [10,11,12,13,14,15,16,17,18,19,20] on concurrent robot-assisted radical prostatectomy (RARP) and robotic inguinal hernia repair (IHR).

II. Concurrent RARP + Robotic Inguinal Hernia Repair (IHR)
  1. Operative Time:
    ○ The operative time reported in the literature ranged from 140.0 to 192.5 min, with additional values being indicated as “+10 over RARP” and “+24 over RARP”. In our study, the average operative time was 221.6 min, slightly exceeding the upper end of this range. This extended duration may be attributed to the complexity and precision required in our surgical procedures (one patient with concomitant locally advanced prostate cancer and three hernia sites), which could involve more intricate steps and careful handling of anatomical structures. The overall certainty of the evidence for operative time was rated as low due to the considerable variability observed across studies (I^2^: 90%). The mean operative time of 172.12 min (95% CI: 155.42–188.83) aligns with reports in the literature, but the very high heterogeneity significantly limits the precision of this estimate.
  2. Estimated Blood Loss:
    ○ The blood loss reported in the studies varied from 50 to 175 mL. Our study documented an average estimated blood loss of 358.3 mL, which was higher than the values reported in the literature. This notable difference may be due to various aforementioned factors, patient comorbidities, and the meticulous recording of intraoperative blood loss in our institution. Further investigation into intraoperative blood management strategies could be beneficial. The certainty of the evidence for estimated blood loss was rated as very low due to the extreme variability observed across studies (I^2^: 99%). The mean estimated blood loss of 143.80 mL (95% CI: 87.37–200.22) shows significant variation between institutions, reducing the confidence in this estimate.
  3. Complications:
    ○ Complications were generally minimal across all studies, with descriptions ranging from “none” to “minor”. In our study, complications were classified as “minor (Grade I–II)”, aligning with the literature and confirming the procedural safety. The low rate of significant complications underscores the efficacy of our surgical technique and postoperative care protocols. The certainty of the evidence for complications was rated as high, with an I^2^ value of 10%, indicating low variability between studies. The mean complication rate of 2.14% (95% CI: 0.03–4.25%) suggests a high level of procedural safety with consistent outcomes across studies.
  4. Recurrence Rate:
    ○ The recurrence rate in the literature ranged from 0% to 11%. Our study noted an absence of recurrences, which was consistent with the best outcomes in the literature. This suggested that our surgical methods were highly effective in achieving durable repairs and preventing recurrence (Figure 7). The certainty of the evidence for recurrence rate was rated as high, with an I^2^ value of 5%, reflecting low variability between studies. The mean recurrence rate of 2.01% (95% CI: 0.04–3.98%) is consistent with favorable outcomes reported in the literature.
  5. Hernia Side:
    ○ The reviewed studies predominantly reported unilateral hernias, with bilateral cases being less common. In our study, we observed two cases of a right-sided hernia and one bilateral case with an additional epigastric (linea alba) hernia. This distribution was in line with the literature, indicating that our patient cohort was representative of the general population undergoing similar procedures.
  6. Patient BMI:
    ○ The average BMI reported in the studies ranged from 26.47 to 28.0 kg/m^2^. Our study did not provide data on the BMI, which limited direct comparison. However, future studies should include the BMI as a variable to better understand its impact on surgical outcomes and to allow for a more comprehensive analysis.
  7. Hospital Stay:
    ○ The length of hospital stay varied from 1 to 6 days in the reviewed studies. Our study reported an average hospital stay of 7 days, slightly exceeding the upper range reported in the literature. This prolonged hospitalization period may reflect our institution’s cautious approach to pre-, intra-, and postoperative care, ensuring complete recovery before discharge in complex cases. Reviewing and optimizing postoperative protocols could potentially reduce the length of stay without compromising patient safety. The certainty of the evidence for hospitalization time was rated as moderate, with an I^2^ value of 60%, indicating substantial variability between institutions. The mean hospitalization time of 3.29 days (95% CI: 1.89–4.69) suggests that variability between centers may affect this metric.
  8. Follow-Up Period:
    ○ The follow-up period in the literature ranged from 9.0 to 36.6 months. Our study had a follow-up period ranging from 15 to 35 months, which was consistent with the literature. Adequate follow-up is crucial for monitoring long-term outcomes and ensuring the durability of surgical repairs.

Summary

Our institutional study demonstrated outcomes largely consistent with those reported in the literature concerning the safety and efficacy of surgical procedures. Although the operative time and blood loss in our cohort were higher than average, the overall certainty of the evidence for these metrics was rated as low due to substantial heterogeneity across studies. Despite these variations, the low recurrence and complication rates reinforce the safety and effectiveness of the procedure, with high certainty of evidence for these outcomes. The slightly prolonged hospital stay suggests a need to review and optimize postoperative care protocols to reduce recovery times. Future efforts should focus on minimizing blood loss and standardizing surgical protocols to further improve recovery times and patient outcomes.

III. Other Concurrent Robotic Multisite Surgery Procedures
  1. Limited Case Volume:
    ○ The surgical interventions discussed, including robot-assisted adrenalectomy (RAA) in conjunction with robot-assisted partial nephrectomy (RAPN) and robot-assisted cystolithotomy (RACLT) performed concurrently with robot-assisted radical prostatectomy (RARP), are rarely performed surgical procedures. Both our institutional data and the existing literature emphasize that these procedures are reserved for selected cases, reflecting their niche status in urological robotic surgery.
  2. Non-Standardized, Individualized Approaches:
    ○ These procedures often represent customized surgical solutions tailored to the unique clinical situations of patients with complex or concurrent pathologies. The inherent variability in these surgical combinations presents a challenge in establishing a standardized protocol or reliably predicting outcomes across different patient populations.
  3. Limitations in Deriving Conclusive Evidence:
    ○ The small number of cases analyzed makes it difficult to derive definitive conclusions regarding the overall safety and efficacy of these interventions. Although the outcomes from both our institution and the broader literature suggest that these surgeries can be performed with a favorable safety profile and minimal complications, the limited scope of available data necessitates cautious interpretation when considering broader applications.
  4. Contextual Understanding and Observational Insights:
    ○ Despite the constraints imposed by the small sample size, certain trends emerged from the data. The operative times, intraoperative blood loss, and complication rates observed in our cases were consistent with those documented in the literature, implying that these complex procedures can yield satisfactory outcomes with meticulous patient selection and surgical precision. However, the slightly prolonged hospital stays in our cohort in comparison with those reported in the literature may indicate a more conservative approach to postoperative care, which should be re-evaluated in order to streamline recovery protocols.

Summary

Although the limited case volume and non-standardized nature of these concurrent robotic multisite surgeries restrict the generalizability of the findings, the similarities in outcomes across various reports and our institutional data provide valuable context. These observations suggest that, with appropriate surgical planning and expertise, even complex and uncommon multisite robotic procedures can be performed successfully, with outcomes that align with established safety and efficacy standards.

## 5. Discussion

I. RARP + RAPN

Feasibility and Safety

The concurrent performance of RARP and RAPN offers a unique approach to managing patients with synchronous primary cancers of the prostate and kidney. The concurrent execution of RARP and RAPN was demonstrated to be feasible and safe across multiple studies. For instance, Boncher et al. reported a mean operative time of 410 min with no perioperative complications in a series of four patients [1]. Similarly, Guttilla et al. observed no significant complications in their study of three patients, with a mean operative time of 390 min [5]. In our institutional experience, both procedures were completed without significant intraoperative complications or conversions to open surgery, with a mean operative time of 315 min and minimal blood loss. This aligns with the findings from Akpinar et al., who reported an average operative time of 400 min and low blood loss [8]. Additionally, Piccoli et al. demonstrated that simultaneous robotic surgeries are feasible, highlighting their efficiency and safety in a larger cohort of seven patients [9].

Oncological Outcomes

Pathological examination of our patients revealed no cases of positive surgical margins following RARP or RAPN, although they are reported in the literature. For example, Jung et al. reported two cases of positive margins in a study of five patients [6]. The importance of achieving negative margins cannot be overstated, as positive margins are associated with a higher risk of cancer recurrence [5,9,26].

Renal Function

Postoperative renal function was closely monitored in our patients, with eGFR measurements being taken several hours and several dozen hours postoperatively. None of our patients experienced a temporary decline in eGFR immediately following surgery. This finding is inconsistent with the transient renal impairment that is often observed after partial nephrectomy due to ischemia–reperfusion injury [7]. Similar findings were also reported by Raheem et al., who observed temporary declines in eGFR that stabilized within a month [2]. Long-term preservation of renal function remains a critical consideration in partial nephrectomy, and our results suggest that concurrent surgery does not significantly compromise renal outcomes [3,4].

Technical Considerations

The use of the da Vinci X robotic system with dual-console capabilities allowed for the efficient execution of both procedures. The need for repositioning and re-docking adds complexity but is manageable with a well-coordinated team. Modifications to trocar placement, as described by Valero et al., facilitate the transition between procedures, optimizing efficiency and minimizing additional incisions [7]. Additionally, Cochetti et al. emphasized the importance of dual-console systems in improving surgical outcomes by allowing the simultaneous participation of two surgeons, thereby enhancing the learning curve and surgical efficiency [3]. Notably, our docking method was not previously described in the literature, and it also uses the dual-console technique.

Limitations

Our study’s limitations include its small sample size, retrospective design, and lack of long-term follow-up. Further research with larger cohorts and prospective data is necessary to validate these findings and refine the surgical approach. Similar recommendations were made in multiple studies, emphasizing the need for larger studies to confirm their findings [2,7,8]. Additionally, long-term follow-up is essential to assess the durability of oncological control and renal function, as highlighted by multiple authors [4,6,9].

II. RARP + RTAPPIHR

Feasibility and Safety

The concurrent performance of RARP and RTAPPIHR proved to be both feasible and safe. Multiple studies confirmed that the addition of IHR during RARP does not significantly increase the operative time or complication rates. For example, Mourmouris et al. reported that the addition of RTAPP IHR only minimally increased the mean console time by 24 min and did not result in significant postoperative complications or hernia recurrences during an average follow-up of 32.1 months [14]. Joshi et al. also demonstrated no postoperative complications or hernia recurrences at a mean follow-up of 34 months [11]. Moreover, Rogers et al. found that combining RARP with IHR did not significantly alter perioperative outcomes. Their study highlighted that simultaneous hernia repair reduced the need for subsequent surgical interventions for hernia repair, demonstrating the safety and efficiency of this combined approach. The median follow-up in their study was 36.6 months, with no mesh-related complications reported, further supporting the safety of this procedure [16].

Outcomes

The outcomes from concurrent RARP and IHR are generally favorable, with low recurrence rates and minimal postoperative complications. Finley et al. reported no hernia recurrences or mesh-related complications in their series of 49 herniorrhaphies performed during RARP [10]. In a similar way, Atmaca et al. found no significant difference in operative time or estimated blood loss between patients undergoing RARP alone and those undergoing concurrent RARP and IHR, with no mesh-related complications during a median follow-up of 13 months [17]. Bajpai et al. demonstrated that the addition of IHR to RARP did not adversely affect perioperative outcomes, including estimated blood loss and hospital stay [19]. Furthermore, a study by Jaber et al. indicated that the combined procedure resulted in low rates of mesh-related complications, reaffirming the positive outcomes associated with this surgical approach [27].

Technical Considerations

Several technical considerations are crucial for optimizing outcomes in concurrent RARP and IHR. Key factors include the type of mesh used and its placement and fixation techniques. Mourmouris et al. used a non-prosthetic, tissue-based technique for direct inguinal hernias involving suturing the lateral edge of the rectus abdominis muscle sheath to the ileopectineal ligament [14]. Atmaca et al. employed various mesh types, such as Ventralight ST and 3DMax, fixed with laparoscopic tackers and absorbable sutures to prevent mesh migration and postoperative complications such as seroma formation and adhesions [17]. Joshi et al. utilized both polypropylene and polyester meshes secured with titanium tacks and closed with a running absorbable suture to ensure effective fixation and minimize the risk of postoperative complications [11]. Bajpai et al. highlighted the importance of using adhesion-resistant meshes to reduce the risk of postoperative complications, particularly given the proximity of the mesh to the vesicourethral anastomosis during the transperitoneal procedure [19].

Limitations

Despite the encouraging results, several limitations must be acknowledged. Most studies on concurrent RARP and IHR are retrospective and involve relatively small sample sizes, limiting the generalizability of their findings. Additionally, longer follow-up periods are needed to fully assess the long-term outcomes and potential complications associated with this combined approach. Soto-Palou et al. emphasized the need for standardized surgical techniques and mesh types to ensure consistent outcomes across different institutions [28].

In conclusion, concurrent RARP and RTAPPIHR is a feasible and safe approach with favorable outcomes. Further research is needed to confirm these findings and refine the surgical techniques and materials used in this combined approach to optimize patient care.

III. Other Concurrent Urological Robotic Multisite Surgery Procedures

Feasibility and Safety

The feasibility of concurrent urological robotic surgery multisite procedures was demonstrated across various studies. Pisipati et al. reported on the safety and technical feasibility of combined upper and lower urinary tract robotic surgery, highlighting acceptable complication rates and good oncological outcomes [29]. Similarly, Ferrari et al. successfully performed the concurrent excision of a retroperitoneal paraganglioma and radical prostatectomy using a robotic approach, with no complications reported, reinforcing the feasibility of these complex procedures [30]. Furthermore, Gul et al. conducted a study on RAPN and robot-assisted adrenalectomy, showing that these concurrent procedures were not only feasible, but also did not result in any significant postoperative complications [24].

The safety outcomes for concurrent robotic urological multisite surgeries are promising. Tan et al. reported no significant complications in patients undergoing RARP and robot-assisted cystolithotomy, highlighting the procedure’s safety [21]. Moreover, Tonooka et al. highlighted that in complex cases such as concurrent RAPN and robot-assisted laparoscopic ileocecal resection for synchronous cancers, a minimally invasive approach reduced surgical risks and facilitated quicker recovery, emphasizing the advantages of concurrent procedures in managing multiorgan medical conditions [31].

Outcomes

The outcomes of concurrent robotic multisite urological surgery procedures are generally favorable, with multiple studies documenting successful results. Tan et al. documented no significant complications in patients undergoing combined RARP and robot-assisted cystolithotomy procedures, with early discharge and improved urinary symptoms [21]. Ferrari et al. emphasized no postoperative complications in concurrent excision of retroperitoneal paraganglioma and radical prostatectomy [30]. Flavin et al. noted the benefits of robotic surgery, such as lower morbidity, improved convalescence, reduced postoperative pain, shorter hospital stays, and better cosmetic outcomes compared with open procedures [32].

Technical considerations

The technical considerations for performing concurrent robotic urological multisite surgeries encompass several critical aspects. Preoperative planning necessitates meticulous imaging and strategic preparation. Manfredi et al. underscored the importance of utilizing 3D augmented-reality models to enhance perioperative outcomes and inform surgical approaches in robot-assisted urologic procedures [33]. Intraoperatively, advanced robotic instruments are essential for achieving precision. Hashira et al. demonstrated the feasibility and safety of a humanoid hand system for robotic urological surgery, providing stable assistance through a small incision port [34]. Zhang et al. highlighted the use of 3D image reconstruction in robotic urological surgery, reporting no conversions to open surgery and minimal blood loss, thus emphasizing the significance of advanced imaging techniques in postoperative management [35].

## 6. Conclusions

Concurrent RARP and RAPN for patients with synchronous prostate cancer and small renal tumors seem to be a feasible and safe approach. Our institutional results demonstrate non-inferior mean operative times, minimal blood loss, and no significant complications, in line with the current literature. Importantly, our findings show a remarkable improvement in postoperative renal function, with an increase in eGFR, further supporting non-inferior functional outcomes. Oncological outcomes were also favorable, with no positive surgical margins being reported in our cases, potentially confirming the precision and effectiveness of this approach.

Our results for concurrent RARP and RTAPPIHR also indicate safety and efficacy, with no major complications and no recurrences of hernias during follow-up. Although the operative time for these combined procedures was slightly longer than that reported in the literature, the low complication rates and absence of mesh-related issues support the potential feasibility of performing both procedures in a single session. This combined approach eliminates the need for future surgical interventions for hernia repair, possibly offering an efficient and patient-centered solution.

The feasibility and safety of other multisite robotic procedures, such as RAPN with adrenalectomy and cystolithotomy with RARP, were also supported by our institutional experience. Although limited by smaller case volumes, these complex surgeries were performed without significant complications, and the postoperative outcomes were in line with the literature. These procedures, while not as common, may highlight the versatility of robotic surgery in managing complex urological cases within a single operative session.

Although no direct cost analysis was conducted, the combined nature of the procedure suggests potential reductions in cumulative operative time and hospitalization, which could translate into cost savings and reduced patient burden.

## 7. Future Directions

The future of concurrent urological robotic multisite surgery procedures is promising, with significant advancements in single-port systems, training programs, and the integration of new technologies [36,37]. These developments are expected to enhance the precision, cost-effectiveness, and accessibility of robot-assisted surgery, ultimately improving patient outcomes and expanding the reach of minimally invasive surgical options in urology.

Future research should focus on prospective multicenter studies with larger patient cohorts to validate the current findings and refine surgical techniques. Investigating the cost-effectiveness of concurrent RARP and RAPN, as well as RARP and RTAPPIHR, in comparison with separate procedures could also provide valuable insights for clinical practice. Additionally, exploring the impact of various mesh types and fixation methods on long-term outcomes and patient quality of life would help optimize the latter procedure.

Smaller healthcare facilities may struggle with the financial and logistical demands associated with the implementation of concurrent urological robotic surgeries [38]. To address these challenges, effective training programs and strategic resource allocation are imperative. Furthermore, continuous technological advancements and robust policy support are crucial for the broader adoption of these surgical techniques. The coordination of multisite surgeries necessitates the intricate integration of various systems and teams, which requires robust real-time communication to mitigate potential errors [39]. Additionally, the training of surgeons presents a significant challenge, as mastering robotic systems necessitates substantial investment in simulation-based programs and ongoing assessment [40]. In summary, the future of concurrent urological robotic surgery hinges on overcoming these multifaceted barriers.

The da Vinci™ system, developed by Intuitive Surgical in Sunnyvale, USA, is a cornerstone of robotic-assisted laparoscopic surgeries, including radical prostatectomies, nephrectomies, and cystectomies. The success of this technology catalyzed the development of several new platforms, such as Versius™ (by CMR Surgical in Cambridge, UK), Hugo™ RAS (by Medtronic in Minneapolis, MN, USA), Senhance™ (by Asensus Surgical in Durham, NC, USA), Avatera™ (by Avateramedical GmbH in Jena, Germany), Revo-I™ (by Meere Company Inc. in Yongin, Republic of Korea), and Hinotori™ (by Medicaroid Inc. in Kobe, Japan), which promise to enhance the versatility, cost-effectiveness, and accessibility of robotic surgical systems [41,42,43,44,45,46,47]. The recently developed single-port robotic surgery systems aim to reduce surgical trauma by operating through a single incision, potentially improving cosmetic outcomes and reducing recovery times and tissue trauma. These systems offer the benefits of minimally invasive surgery while overcoming the limitations associated with traditional multi-port systems, such as increased complexity and longer operative times [46]. The integration of artificial intelligence, machine learning, and augmented reality into robotic systems could enhance precision, reduce errors, and provide real-time assistance to surgeons [47,48,49]. Increased competition and technological advancements are expected to drive down costs, making these systems more accessible to a broader range of healthcare providers and patients [46]. Proctoring, whether in person or remotely, positively impacts surgical performance, particularly in intermediate tasks. This approach can significantly enhance training and proficiency, making robotic surgery more accessible globally [50].

## Figures and Tables

**Table 1:** Newcastle–Ottawa Scale scores for studies on robot-assisted radical prostatectomy (RARP) + robot-assisted partial nephrectomy (RAPN).

Study	Selection	Comparability	Outcome	Total
Patel et al. (2009) [4]	4	2	3	9
Boncher et al. (2010) [1]	4	2	3	9
Guttilla et al. (2011) [5]	3	2	3	8
Jung et al. (2012) [6]	3	2	3	8
Raheem et al. (2016) [2]	4	2	3	9
Valero et al. (2017) [7]	3	2	3	8
Akpinar et al. (2019) [8]	4	2	3	9
Cochetti et al. (2020) [3]	3	2	3	8
Piccoli et al. (2021) [9]	4	2	3	9

**Table 2:** Newcastle–Ottawa Scale scores for studies on robot-assisted radical prostatectomy (RARP) + robotic transabdominal preperitoneal inguinal hernia repair (RTAPPIHR).

Study	Selection	Comparability	Outcome	Total
Finley et al. (2007) [10]	4	2	3	9
Joshi et al. (2010) [11]	4	2	3	9
Kyle et al. (2010) [12]	3	2	3	8
Lee et al. (2013) [13]	3	2	3	8
Mourmouris et al. (2016) [14]	4	2	3	9
Ludwig et al. (2016) [15]	3	2	3	8
Rogers et al. (2017) [16]	4	2	3	9
Atmaca et al. (2018) [17]	3	2	3	8
Xia et al. (2018) [18]	4	2	3	9
Bajpai et al. (2020) [19]	4	2	3	9
Bedir et al. (2021) [20]	3	2	3	8

**Table 3:** Newcastle–Ottawa Scale scores for other corresponding concurrent urological robotic multisite surgery procedures.

Study	Selection	Comparability	Outcome	Total
Tan, G.Y. et al. (2012) [21]	4	2	3	9
Macedo, F.I.B. et al. (2013) [22]	3	1	2	6
Sappal, S. et al. (2016) [23]	3	2	3	8
Gul, Z.G. et al. (2020) [24]	4	2	3	9
Olive, E.J. et al. (2024) [25]	4	1	2	7

**Table 4:** Detailed outcomes for institutional cases of concurrent robot-assisted radical prostatectomy (RARP) + robot-assisted partial nephrectomy (RAPN). * Estimated glomerular filtration rate.

Patient Number	1 (of 4)	2 (of 4)
Operation Date	30 June 2023	13 December 2023
Hospitalization Period	27 June 2023–4 July 2023 (7 days in total, 4 days postoperatively)	12–16 December 2023 (4 days in total, 3 days postoperatively)
Preoperative Hemoglobin (mmol/L)	8.9	9.4
Postoperative Hemoglobin (mmol/L)	8.3	9.1
Preoperative eGFR * (mL/min/1.73 m^2^)	66.1	73.6
Postoperative eGFR * (mL/min/1.73 m^2^)	99.5	89.9
Preoperative Prostate Cancer Clinical Stage and Biopsy Result	Adenocarcinoma Gleason 8 (4 + 4) ISUP Grade 4 cT2bN0M0	Adenocarcinoma Gleason 7 (3 + 4) ISUP Grade 2 cT2bN0M0
Post-Prostatectomy Stage and Histopathology Result	Adenocarcinoma Gleason 8 (4 + 4) ISUP Grade 4 pT2cN0M0R0 5 lymph nodes dissected during extended pelvic lymph node dissection	Adenocarcinoma Gleason 7 (3 + 4) ISUP Grade 2 pT2cN0M0R0 without end
Preoperative Kidney Tumor Clinical Stage and Side	cT1a solid tumor right side, upper pole	cT2a cystic tumor Bosniak III right side, posterior aspect
Postoperative Kidney Tumor Histopathology Result	Oncocytoma without involvement of surrounding fatty tissue R0	pT0 Necrotic connective tissue fragments in the form of a cyst; neoplastic tissue was not found
Total Operative Time (min)	345	285
Console Time (min)	RAPN: 105 Repositioning: 50 RARP + extended pelvic lymph node dissection: 190	RAPN: 65 Repositioning: 30 RARP: 180
Estimated Blood Loss (mL)	350	250
Complications (Clavien–Dindo)	None	None

**Table 5:** Detailed outcomes for institutional cases of multitemporal robot-assisted radical prostatectomy (RARP) + robot-assisted partial nephrectomy (RAPN). * Estimated glomerular filtration rate.

Patient Number	3 (of 4)	4 (of 4)
Operation Date	RARP (2nd) 11.09.2023; RAPN (1st) 05.07.2023; extensive adhesions in the peritoneal cavity, perirenal “toxic fat”	RARP + extended pelvic lymph node dissection 13 February 2024; RAPN 27 May 2024
Hospitalization Period	RARP (2nd) 06.09.2023–15.09.2023 (9 days in total, 4 days postoperatively); RAPN (1st) 28.06.2023–09.07.2023 (11 days in total, 4 days postoperatively)	RARP + extended pelvic lymph node dissection (1st) 12–17 February 2024 (5 days in total, 4 days postoperatively); RAPN (2nd) 26–31 May 2024 (5 days in total, 4 days postoperatively)
Preoperative Hemoglobin (mmol/L)	RARP 8.5 RAPN 8.5	RARP + extended pelvic lymph node dissection 8.4 RAPN 8.8
Postoperative Hemoglobin (mmol/L)	RARP 6.9 RAPN 7.7	RARP + extended pelvic lymph node dissection 7.6 RAPN 7.9
Preoperative eGFR * (mL/min/1.73 m^2^)	RARP 96.5 RAPN 93.6	RARP + extended pelvic lymph node dissection 88.9 RAPN 79.1
Postoperative eGFR * (mL/min/1.73 m^2^)	RARP 95.6 RAPN 87.4	RARP + extended pelvic lymph node dissection 63.6 RAPN 72.3
Preoperative Prostate Cancer Clinical Stage and Biopsy Result	Adenocarcinoma Gleason 7 (3 + 4) ISUP Grade 2 cT2bN0M0	Adenocarcinoma Gleason 7 (4 + 3) ISUP Grade 3 cT3N1M0 locally advanced tumor, preoperative PSA 64 ng/mL
Post-Prostatectomy Stage and Histopathology Result	Adenocarcinoma Gleason 7 (3 + 4) ISUP Grade 2 pT2cN0M0R0 without extended pelvic lymph node dissection	Adenocarcinoma Gleason 8 (4 + 4) ISUP Grade 4 pT2cN0M0 7 lymph nodes dissected during extended pelvic lymph node dissection
Preoperative Kidney Tumor Clinical Stage and Side	cT1a solid tumor left side, upper pole	cT1b cystic tumor Bosniak III left side, posterior aspect
Preoperative Kidney Tumor Histopathology Result	pT0 Histo-oncological changes in the examined material were absent	Papillary renal cell carcinoma, type 1, G2). The tumor mass showed foci of necrosis and congestion. pT1bR0
Total Operative Time (min)	RAPN 210 RARP 225	RAPN 230 RARP + extended pelvic lymph node dissection 230
Console Time (min)	RAPN 170 RARP 205	RAPN 200 RARP + extended pelvic lymph node dissection 195
Estimated Blood Loss (mL)	RAPN 350 RARP 550	RAPN 300 RARP 250
Complications (Clavien–Dindo)	Grade II Fever, treated with antibiotic therapy	None

**Table 6:** Detailed outcomes for institutional cases of concurrent robot-assisted radical prostatectomy (RARP) + robotic transabdominal preperitoneal inguinal hernia repair (RTAPPIHR). * Estimated glomerular filtration rate.

Patient Number	1 (of 3)	2 (of 3)	3 (of 3)
Operation Date	17 August 2021	20 October 2021 + extended pelvic lymph node dissection	9 February 2023 + extended pelvic lymph node dissection
Hospitalization Period	11–20 August 2021 (9 days in total, 3 days postoperatively)	18–25 October 2021 (7 days in total, 5 days postoperatively)	8–13 February 2023 (5 days in total, 4 days postoperatively)
Preoperative Hemoglobin (mmol/L)	9.8	9.7	8.6
Postoperative Hemoglobin (mmol/L)	N/A	9.2	5.9
Preoperative eGFR * (mL/min/1.73 m^2^)	92.2	81	66.3
Postoperative eGFR * (mL/min/1.73 m^2^)	N/A	N/A	82.5
Preoperative Prostate Cancer Clinical Stage and Biopsy Result	Adenocarcinoma Gleason 6 (3 + 3) ISUP Grade 1 cT2aN0M0	Adenocarcinoma Gleason 7 (3 + 4) ISUP Grade 2 cT3N0M0	Adenocarcinoma Gleason 7 (3 + 4) ISUP Grade 2 cT2bN0M0
Post-Prostatectomy Stage and Histopathology Result	Adenocarcinoma Gleason 6 (3 + 3) ISUP Grade 1 pT2aN0MxR0	Adenocarcinoma Gleason 7 (4 + 3) ISUP Grade 3 pT3aN0MxR0	Adenocarcinoma Gleason 8 (4 + 4) ISUP Grade 4 pT3bN0MxR1 single-point positive surgical margin
Hernia Side	RIGHT SIDE	RIGHT SIDE	BOTH SIDES + EPIGASTRIC HERNIA (LINEA ALBA)
Total Operative Time (min)	195	200	270
RARP Console Time (min)	135 Repositioning: not necessary	165 Repositioning: not necessary	155 Repositioning: not necessary
Inguinal Hernia Repair Console Time (min)	25	40	65
Estimated Blood Loss (mL)	150	225	700
Complications (Clavien–Dindo)	Grade I SARS-CoV-2 infection requiring antipyretics	NONE	Grade II red cell concentrate transfusion

**Table 7:** Detailed outcomes for institutional cases of other concurrent robotic multisite surgery procedures. * Estimated glomerular filtration rate.

Patient Number	1 (of 3)	2 (of 3)	3 (of 3)
Operation Date and Type of Robotic Procedure	8 May 2023 RAPN + IPSILATERAL ROBOT-ASSISTED ADRENALECTOMY (RAA)	27 June 2023 ROBOT-ASSISTED TOTAL TRANS-OBTURATOR TAPE (TOT) REMOVAL + ROBOT-ASSISTED CYSTOLITHOTOMY (RACLT)	29 December 2023 RARP + ROBOT-ASSISTED CYSTOLITHOTOMY (RACLT)
Indication	cT4N0M0 PERIPHERAL UPPER POLE KIDNEY TUMOR WITH ADRENAL INVOLVEMENT	INTRAVESICAL TRANS-OBTURATOR TAPE (TOT) EROSION WITH CONCOMITANT BLADDER STONE	ORGAN-CONFINED PROSTATE CANCER WITH CONCOMITANT BLADDER STONE
Hospitalization Period	7–15 May 2023 (8 days in total, 7 days postoperatively)	27 June 2023–4 July 2023 (7 days in total, 7 days postoperatively)	28 December 2023–3 January 2024 (6 days in total, 5 days postoperatively)
Pre-operative Hemoglobin (mmol/L)	8.6	7.5	8.7
Postoperative Hemoglobin (mmol/L)	8	6.5	8.2
Preoperative eGFR * (mL/min/1.73 m^2^)	107.7	96.1	98.1
Postoperative eGFR * (mL/min/1.73 m^2^)	123.2	82.9	103.6
Preoperative Prostate Cancer Clinical Stage and Biopsy Result	N/A	N/A	Adenocarcinoma Gleason 6 (3 + 3) ISUP Grade 1 cT2aN0M0
Post-Prostatectomy Stage and Histopathology Result	N/A	N/A	Adenocarcinoma Gleason 9 (4 + 5) ISUP Grade 5 pT2cN0M0R0
Postoperative Kidney Tumor Histopathology Result	Clear cell renal cell carcinoma (RCC Fuhrman 2 WHO G2 R0	N/A	N/A
Total Operative Time (min)	265	125	165
Console Time (min)	230 Repositioning: not necessary	100 Repositioning: not necessary	130 Repositioning: not necessary
Estimated Blood Loss (mL)	800	250	200
Complications (Clavien–Dindo)	Grade II red cell concentrate transfusion	None	None

**Table 8:** Summary of cases of concurrent robot-assisted radical prostatectomy (RARP) + robot-assisted partial nephrectomy (RAPN) from the literature in comparison with the authors’ own (institutional) surgical results (chronological order). * Estimated glomerular filtration rate.

Study (Year of Surgery)	Country	Number of Patients	Robotic System	Institution	Mean Operative Time (min)	Mean Console Time (min)	Mean Estimated Blood Loss (mL)	Complications (Clavien–Dindo)	Positive Surgical Margins	Mean Preoperative eGFR * (mL/min/1.73 m^2^)	Mean Preoperative eGFR * (mL/min/1.73 m^2^)	Calculated Mean Difference in eGFR * before and after Surgery (mL/min/1.73 m^2^)	Follow-Up	Mean Hospitalization Time (Days)
Patel et al. (2009) [4]	USA	1	da Vinci S	Henry Ford Hospital	427	335	200	None	None	N/A 1.1 mg/dL creatinine level	N/A	N/A	4 months	2
Boncher et al. (2010) [1]	USA	4	da Vinci S	Michigan State University	410	270	310	None	1	82	78	−4	1 month	7
Guttilla et al. (2011) [5]	Italy	3	da Vinci S	University of Padua	390	250	290	None	0	83	78	−5	1 month	6
Jung et al. (2012) [6]	Republic of Korea	5	da Vinci Si	Seoul National University Hospital	415	275	305	None	2	84	79	−5	1 month	7
Raheem et al. (2016) [2]	Republic of Korea	6	da Vinci Xi	Yonsei University College of Medicine	395	255	315	None	2	80	75	−5	1 month	5
Valero et al. (2017) [7]	USA	3	da Vinci Xi	Cleveland Clinic	405	265	300	None	1	85	80	−5	1 month	6
Akpinar et al. (2019) [8]	Turkey	5	da Vinci Xi	Istanbul University	400	260	300	None	1	85	80	−5	1 month	7
Cochetti et al. (2020) [3]	Italy	6	da Vinci Xi	University of Perugia	420	280	320	None	1	80	75	−5	1 month	8
Piccoli et al. (2021) [9]	Brazil	7	da Vinci Xi	Hospital de Clínicas de Porto Alegre	430	290	330	None	1	86	82	−4	1 month	7
Our work	Poland	2	da Vinci X	Multidisciplinary Hospital in Warsaw-Miedzylesie	315	270	300	None	0	69.85	94.7	+24.85	12 months	5.5

**Table 9:** Summary of concurrent robot-assisted radical prostatectomy (RARP) + robot-assisted inguinal hernia repair (IHR) cases from the literature in comparison with the authors’ own surgical results (chronological order).

Author (Year of Study)	Center (Country)	Number of Surgeries	Number of Hernia Repairs	Repair Method	Mean Operative Time (min)	Blood Loss (mL)	Complications (Clavien–Dindo)	Follow-Up (Months)	Recurrence Rate	Hernia Side (Left—L/Right—R/Bilateral—B)	Average BMI (kg/m^2^)	Hospital Stay (Days)	Years of Surgeries—Time Frame
Finley et al. (2007) [10]	University of California-Irvine (USA)	533	49	Mesh	+10 over RARP	N/A	None	15.3	2%	31 L 9 R	N/A	N/A	2002–2006
Joshi et al. (2010) [11]	North Shore University Hospital, NY (USA)	4	6	Mesh	+24 over RARP	N/A	None	34	0%	2 L 2 R 2 B	N/A	N/A	2008–2009
Kyle et al. (2010) [12]	Royal Melbourne Hospital (Australia)	700	37	Mesh	+5–10 over RARP	N/A	None	29	2.7%	18 L 14 R 5 B	27.1	2	2005–2009
Lee et al. (2013) [13]	University of Iowa, IA (USA)	1118	91	Mesh	185	170	1 recurrence, others not significant	9–12	1.1%	41 L 29 R 22 B	27.5	1	2010–2012
Mourmouris et al. (2016) [14]	Acibadem Maslak Hospital, Istanbul (Turkey)	1005	29	Nonprosthetic	147	175	None	32.1	0%	7 L 14 R 8 B	26.47	4,3	2013–2015
Ludwig et al. (2016) [15]	University of Pittsburgh Medical Center (USA)	71	11	Mesh	160	100	Minor	36	0%	5 L 3 R 3 B	27.0	2	2010–2014
Rogers et al. (2017) [16]	Florida Hospital, Celebration, FL (USA)	1139	39	Mesh transabdominal preperitoneal (TAPP)	188	110.87	10.26% Minor	N/A	0%	N/A	26.8	N/A	2008–2015
Atmaca et al. (2018) [17]	Health Sciences University (Turkey)	100	38	Mesh	160	50	7% (Minor)	36.6	11%	19 L 12 R 7 B	N/A	N/A	2014–2017
Xia et al. (2018) [18]	Johns Hopkins University School of Medicine (USA)	198	25	Mesh	155	110	None	20	1.5%	10 L 8 R 7 B	27.3	2	2015–2017
Bajpai et al. (2020) [19]	Miami Cancer Institute (USA)	104	35	Mesh	140	120	None	18	2%	15 L 12 R 8 B	27.5	2	2017–2019
Bedir et al. (2021) [20]	Erzurum Regional Training Hospital (Turkey)	26	32	Mesh	192.5	100	None	18	3.8%	10 L 11 R 5 B	28.0	6	2018–2020
Our work	Multidisciplinary Hospital in Warsaw-Miedzylesie (Poland)	241	3	Mesh transabdominal preperitoneal (TAPP)	221.6 151.6 mean RARP console time 43.3 mean IHR console time	3583	Minor (Grade I–II)	15–35	0%	2 R 1 B	N/A	7 (4 postoperative days)	2021–2024

**Table 10:** Summary of cases of other corresponding concurrent robotic multisite surgery procedures from the literature (chronological order).

Author	Year	Institution	Country	Procedure	Condition	Discharge Day	Surgery Time	Console Time	Complications
Tan, G.Y. et al. [21]	2012	Weill Cornell Medical College	USA	Robotic-assisted radical prostatectomy and cystolithotomy	Prostate cancer with bladder stones	Postoperative day 1 discharge	N/A	N/A	None
Macedo, F.I.B. et al. [22]	2013	James Buchanan Brady Foundation	USA	Robotic removal of eroded vaginal mesh into the bladder	Vaginal mesh erosion with bladder involvement	N/A	N/A	N/A	None
Sappal, S. et al. [23]	2016	Virginia Commonwealth University	USA	Robotic-assisted partial nephrectomy and adrenalectomy	Intrarenal adrenocortical adenoma	Postoperative day 1 discharge	2 h 10 min	14 min	None
Gul, Z.G. et al. [24]	2020	Icahn School of Medicine at Mount Sinai	USA	Robotic-assisted partial nephrectomy and adrenalectomy	Pheochromocytoma	Postoperative day 1 discharge	N/A	N/A	None
Olive, E.J. et al. [25]	2024	Mayo Clinic	USA	Robotic-assisted intravesical mesh excision	Intravesical mesh erosion with stone	Postoperative day 1 discharge	N/A	N/A	None

## Data Availability

Data are exclusively from the system of the Multidisciplinary Hospital in Warsaw-Miedzylesie. Public access to data is not possible and is not permitted for data protection reasons. However, anonymous patient records, as presented in the tables, may be available from the corresponding author upon reasonable request, subject to institutional and ethical approvals.
